# Development and psychometric evaluation of the Work-Family Enrichment Scale for married shift-working nurses in Korea: a methodological study

**DOI:** 10.4069/whn.2026.02.28

**Published:** 2026-03-31

**Authors:** Mi-Jin Park, Il-Ok Kim

**Affiliations:** 1 Sahmyook Seoul Hospital, Seoul, Korea; 2 College of Nursing, Sahmyook University, Seoul, Korea

**Keywords:** Factor analysis, statistical, Nurses, Shift work schedule, Work-life balance

## Abstract

**Purpose:**

This study aimed to develop and validate the Work-Family Enrichment Scale for married shift-working nurses (WFES-N).

**Methods:**

A methodological study design was used. Preliminary items were generated based on the work-family enrichment theory proposed by Greenhaus and Powell and were refined through a literature review, in-depth interviews, and content validity testing. Data were collected from married shift-working nurses and analyzed using exploratory and confirmatory factor analyses. Reliability and validity, including convergent, discriminant, and criterion validity, were examined.

**Results:**

Exploratory factor analysis of the preliminary items identified five factors that explained 58.6% of the total variance. Subsequent confirmatory factor analysis supported a four-factor structure consisting of 18 items: benefits of shift work, benefits of multiple roles, economic benefits, and benefits of working. The overall model demonstrated good fit indices. Cronbach’s α for the total scale was .93, and test-retest reliability, assessed using the intraclass correlation coefficient, was .95. Convergent validity was supported, with composite reliability ranging from .81 to .92 and average variance extracted values ranging from .59 to .62. Discriminant validity was confirmed because the confidence intervals of the inter-factor correlations did not include 1.00. Criterion validity was established through a significant correlation with the Work-Family Enrichment Scale (r=.76, *p*<.001).

**Conclusion:**

The developed WFES-N demonstrated satisfactory reliability and validity and provides a context-sensitive tool for assessing work-family enrichment among married shift-working nurses. This instrument may be useful in research and practice aimed at promoting nurses’ well-being and work-family balance.

## Introduction

The labor force participation of married healthcare professionals has emerged as an important strategy for addressing low birth rates and population aging, and the employment rate of married women in South Korea increased steadily from approximately 52% in 2016 to 60% in 2022 [1]. This demographic shift contributes to economic independence and improved social status while also increasing social interest in the quality of “work-family interactions” that arise as individuals fulfill multiple roles at work and at home [2]. Accordingly, discussions surrounding the labor participation of married workers have expanded beyond the traditional view of the work-family relationship as one defined only by conflict and now increasingly emphasize the positive interactions between the two domains.

Work-family enrichment is a concept based on the perspective that the work and family domains can exert complementary, positive effects on one another [3-6]. Greenhaus and Powell [7] conceptualized work-family enrichment as a process through which resources generated in work and family roles are transferred across domains. They proposed a theoretical framework suggesting that positive resources developed at work, such as job satisfaction, a sense of accomplishment, and skill acquisition, can improve the quality of family life, whereas emotional support and satisfaction derived from family life can also positively transfer to work. Subsequent studies grounded in this theoretical framework have empirically shown that work-family enrichment may positively influence quality of life and job-related outcomes [5,8-10]. This perspective contrasts with earlier work-family conflict research, which viewed the two domains primarily as competing demands, and instead emphasizes that work and family can strengthen one another [11-13].

Nurses are essential personnel in the healthcare system and provide indispensable medical services. Shift-working nurses, in particular, are responsible for patient health and safety by delivering continuous 24-hour care [14]. Although shift work imposes physical and psychological burdens because of irregular schedules and high work intensity [15,16], its irregular working hours, relative to day work, and its comparatively predictable shift patterns may also allow individuals to restructure their daily routines [14]. For married shift-working nurses who simultaneously perform family roles, these work characteristics may provide a basis for more flexible adjustment of the time and energy available for family life [14]. Previous studies have reported that predictable shift schedules help nurses plan household responsibilities and child-rearing more systematically and may provide opportunities for recovery through more efficient allocation of work and rest [17]. In addition, the distinctive temporal structure of shift work may facilitate role sharing among family members and thereby contribute to more cooperative and positive family relationships [14].

Thus, married shift-working nurses are a group likely to experience not only work-family conflict in an irregular work environment but also work-family enrichment, in which the two domains positively influence one another [14]. However, measurement tools that can systematically capture these positive experiences within the context of shift work and nursing practice remain limited. Although various instruments have been developed internationally to conceptualize and measure work-family enrichment [18-23], most were designed primarily for general office workers in Western societies and do not adequately reflect the nonstandard schedules and fluctuations in daily routines associated with shift work [24,25]. In nurses who provide 24-hour patient care, job characteristics include a combination of workload intensity, professional responsibility, emotional labor, and physical burden [14]. Despite the likelihood that these characteristics influence the formation and transfer of work-family resources, they have not been sufficiently considered in existing instruments.

Several attempts have also been made in South Korea to measure work-family enrichment. Kim et al. [24] validated Carlson et al.’s [18] Work-Family Enrichment Scale (WFES) among married working women in Korea, whereas Yang and Lee [25] and Jang and Jeong [26] developed instruments to measure the positive effects of multiple roles or work-family transfer in working mothers and dual-income couples, respectively. However, these instruments were developed primarily for general married working women or family units [24-26] and therefore have limitations in fully capturing the work-family experiences of married nurses, whose lives reflect both the distinctive conditions of shift work and the professional demands of nursing. These limitations underscore the need to develop a work-family enrichment instrument specifically for married shift-working nurses that reflects this dual context.

Accordingly, this study aimed to develop the Work-Family Enrichment Scale for married shift-working nurses (WFES-N) and to evaluate its reliability and validity. Distinct from the existing WFES, the WFES-N reflects the work and life contexts of married shift-working nurses and provides a foundation for systematically assessing their level of work-family enrichment. The instrument may also serve as evidence to support human resource management and improvements in working conditions in healthcare institutions.

## Methods

**Ethics statement:** This study was approved by the Institutional Review Board of Sahmyook University (No. SYU 2023-12-010-005). Informed consent was obtained from the participants.

### Study design

This methodological study was conducted to develop a WFES-N and to evaluate its reliability and validity.

### Instrument development phase

**Figure 1.**
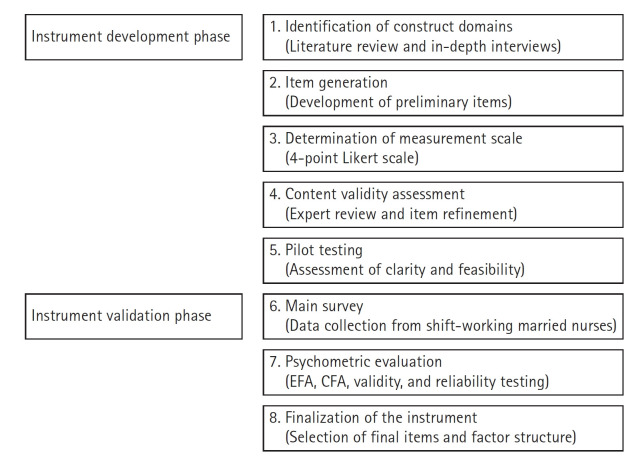
Process of development and validation of the work-family enrichment scale. CFA: Confirmatory factor analysis; EFA: exploratory factor analysis.

This study followed the instrument development and validation procedures proposed by DeVellis [27] and was conducted in two phases: instrument development and instrument validation (Figure 1).

In the instrument development phase, the concepts and key constructs underlying work-family enrichment in married shift-working nurses were derived through a literature review and analysis of in-depth interview data. Based on these findings, the initial pool of preliminary items was generated, and the final preliminary items were selected through content validity assessment and pilot testing.

In the instrument validation phase, the main survey was conducted to evaluate the construct validity, criterion validity, and reliability of the developed instrument, and the final scale was then established.

#### Conceptual framework

This study constructed a conceptual framework to objectively and systematically evaluate work-family enrichment in married shift-working nurses, based on the work-family enrichment theory proposed by Greenhaus and Powell (Supplementary Figure 1) [7]. This theory explains how resources generated in work and family roles are transferred across domains to improve quality of life and describes two pathways of resource transfer: an instrumental pathway, involving skills, knowledge, and time management, and an affective pathway, involving the transfer of positive emotions [7].

This theoretical perspective was applied to the work and life context of married shift-working nurses, deriving a conceptual framework through a literature review and analysis of previous in-depth interview studies (n=10) [14]. According to previous qualitative research, although shift-working married nurses experience role burden and conflict due to their work characteristics, they also have positive experiences where competencies and emotional resources developed through work are transferred to family life. In addition, family support and the relative flexibility of shift work were identified as major factors facilitating work-family balance [14]. In the present study, these work-family enrichment-related contexts were examined in depth and integrated with the literature review findings to derive the core constructs explaining work-family enrichment in married shift-working nurses.

#### Item generation

Based on the attributes of work-family enrichment identified through the literature review and in-depth interviews, a total of 88 preliminary items were generated for married shift-working nurses. After reviewing items with overlapping or similar meanings, 31 items were deleted, leaving 57 preliminary items. These items comprised 15 items on “benefits of working,” 14 on “benefits of family life,” 10 on “benefits of multiple roles,” 11 on “benefits of shift work,” and 7 on “economic benefits.” All items were developed to reflect the conceptual characteristics of each category while also considering item conciseness and ease of comprehension. To minimize central tendency bias and clarify response direction [28], the instrument used a 4-point Likert scale. Each item was scored from 1 (strongly disagree) to 4 (strongly agree), with higher scores indicating greater work-family enrichment among married shift-working nurses.

#### Content validity assessment

For the preliminary items, content validity was assessed twice by a panel of nine academic and clinical experts, based on Lynn’s [28] recommended criterion of 3 to 10 experts.

The first content validity assessment was conducted from September 6 to 12, 2024. The panel comprised two nursing professors; two doctoral-level experts with experience in instrument development and more than 20 years of clinical experience; two married nurses with more than 20 years of clinical experience who were enrolled in doctoral programs; and three doctoral researchers who were married shift-working nurses and had completed training related to instrument development. After the study purpose and conceptual basis of the items were explained, the experts evaluated the relevance of each item using a 4-point Likert scale. The item-level content validity index (I-CVI) ranged from .33 to 1.00, and the scale-level content validity index based on the average method (S-CVI/Ave) was .90. Eight items with an I-CVI below .80 and five items with overlapping meanings or unclear wording were deleted. Reflecting the experts’ feedback, four items were added and one item was revised, resulting in a total of 49 items.

The second content validity assessment was conducted from September 13 to 18, 2024. The original panel of seven experts was expanded to nine by adding one nursing professor and one public health professor. The assessment procedure was identical to that used in the first round. The I-CVI ranged from .67 to 1.00, and the S-CVI/Ave was .90. Three items in the “benefits of working” domain and one item in the “benefits of family life” domain with an I-CVI below .78 were deleted. In addition, three items in the “economic benefits” domain were removed because of redundancy and the potential to confuse respondents. In contrast, items in the “benefits of multiple roles” and “benefits of shift work” domains were retained based on their conceptual importance and support from previous studies. Accordingly, the number of items was reduced from 49 to 42 after the second assessment.

Overall, the 88 preliminary items derived from the literature review and in-depth interviews were refined for overlap and similarity in meaning and then subjected to two rounds of content validity assessment, resulting in a final set of 42 items.

#### Pilot study

In accordance with DeVellis’ [27] recommendation of 20 to 40 participants for pilot testing, a pilot study was conducted from September 25 to 30, 2024, with 23 married shift-working nurses recruited through convenience sampling from tertiary hospitals, general hospitals, and long-term care hospitals in Seoul and Gyeonggi-do. The pilot study was conducted as an online survey. After the study purpose and participation procedures were explained, a survey link was distributed, and participants completed the questionnaire in a self-report format. The pilot study was performed to assess item comprehension, item length and difficulty, font size, questionnaire layout, and response time. The time required to complete the questionnaire ranged from 6 to 15 minutes. Most respondents rated the items as generally easy, and no concerns were reported regarding the questionnaire layout or font size. In addition, the vocabulary and grammatical accuracy of the items were reviewed and revised in consultation with a Korean language scholar. Based on the pilot study findings, the final 42 preliminary items were retained: 7 items on “benefits of working,” 10 on “benefits of family life,” 9 on “benefits of multiple roles,” 9 on “benefits of shift work,” and 7 on “economic benefits.”

### Participants

The main survey was conducted to evaluate the validity and reliability of the final items selected after the pilot study. Participants were recruited through convenience sampling from married shift-working nurses employed at 13 medical institutions nationwide, including three tertiary hospitals, six general hospitals, and four long-term care hospitals. The sample size was determined based on recommended criteria for instrument development studies involving factor analysis. In such studies, exploratory factor analysis (EFA) generally requires a sample size of at least 5 to 10 times the number of items [27], whereas confirmatory factor analysis (CFA) requires at least 200 to 400 participants for stable model estimation [29]. Given the 42 preliminary items, the target sample size was set at a minimum of 420, corresponding to approximately 10 participants per item. To account for potential attrition due to missing or inappropriate responses, the recruitment target was increased to 450. A total of 421 participants completed the survey; after excluding 11 duplicate or ineligible responses, 410 responses were included in the final analysis. Eligible participants were married nurses who had at least 1 year of shift-work experience, at least 1 year of employment at their current workplace, and at least 1 year of marriage, and who voluntarily consented to participate in the study. Nurses with less than 1 year of work experience at their current workplace were excluded to allow for a minimum adaptation period during which a stable pattern of interaction between shift work and family life could develop. This criterion was intended to minimize the influence of unstable life patterns during the early adaptation period and to allow a more stable assessment of work-family enrichment.

### Measurements

The questionnaire used in this study consisted of the final items selected through content validity assessment and pilot testing. It included items from the instrument developed in this study (WFES-N) to measure work-family enrichment in married shift-working nurses, items for criterion validity testing, and items on general characteristics. For criterion validity, the WFES originally developed by Carlson et al. [18] and translated into Korean by Kim et al. [24] was used. This instrument consists of 18 items across two subdomains: work-to-family enrichment (development, affect, and capital) and family-to-work enrichment (development, affect, and efficiency). It uses a 5-point Likert scale, with higher scores indicating greater work-family enrichment. The reliability of the instrument (Cronbach’s α) was .92 in the original study [18], .93 in the Korean validation study [24], and .94 in the present study. In addition, to assess stability, a test-retest evaluation was conducted with 30 married shift-working nurses at a 2-week interval, and the intraclass correlation coefficient (ICC) was calculated [30].

### Data collection

Data were collected through an online survey conducted from October 1 to 8, 2024, among married nurses engaged in shift work at tertiary hospitals, general hospitals, and long-term care hospitals located in Seoul and the metropolitan area (Incheon and Suwon) and in the Yeongnam region (Busan, Gyeongnam, and Daegu). Participants were recruited by distributing a link to the online survey. On the first screen of the survey, an informed consent form was presented that described the study purpose and procedures, confidentiality and anonymity, and the prohibition of using the data for purposes other than the study. The survey was configured so that responses could be submitted only after participants selected the item indicating consent to participate. As a token of appreciation, participants received a small gift equivalent to a mobile coffee coupon worth approximately Korean won 5,000.

### Data analysis

The collected data were analyzed using IBM SPSS ver. 27.0 and AMOS ver. 20.0 (IBM Corp., Armonk, NY, USA). First, participants’ general characteristics were analyzed using descriptive statistics, including frequencies, percentages, means, and standard deviations. Homogeneity between the subsamples used for EFA and CFA was tested using the independent t-test and the chi-square test. Second, construct validity was evaluated through item analysis, EFA, CFA, convergent validity, and discriminant validity. Item analysis included the mean, standard deviation, skewness, kurtosis, item-total correlation coefficient, and changes in reliability after item deletion. The suitability of the data for EFA was assessed using a Kaiser-Meyer-Olkin (KMO) value of at least .60 and a statistically significant Bartlett test of sphericity (*p*<.05), and factor loadings of .40 or greater were considered acceptable for interpretation [31,32]. CFA was performed using maximum likelihood estimation. Model fit was evaluated using the chi-square test, *χ*^2^/degree of freedom (df), the standardized root mean square residual (SRMR), the root mean square error of approximation (RMSEA), the comparative fit index (CFI), the Tucker-Lewis index (TLI), and the incremental fit index (IFI) [33,34]. The criteria for acceptable model fit were *χ*^2^/df ≤3.0, CFI/TLI/IFI ≥.90, RMSEA ≤.08, and SRMR ≤.08 [35-37]. Convergent validity was evaluated using factor loadings of at least .70, an average variance extracted (AVE) of at least .50, and composite reliability (CR) of at least .70. Discriminant validity was confirmed by examining whether the confidence intervals of inter-factor correlation coefficients included 1.00 [38]. Third, criterion validity was assessed by calculating the Pearson correlation coefficient between the instrument developed in this study and the existing WFES. Fourth, internal consistency reliability was evaluated using Cronbach’s α, and test-retest reliability was assessed by calculating the ICC [30].

## Results

Among the 410 participants, the vast majority were female (398, 97.1%). The mean age was 34.65 ±6.30 years, and participants aged 30 to 40 years constituted the largest age group (58.5%). Regarding education, 90.2% held a bachelor’s degree and 9.8% held a graduate degree. In terms of position, staff nurses accounted for the largest proportion (77.1%). Furthermore, 80.0% had less than 10 years of nursing experience. Most participants worked in general wards (88.5%). The mean number of weekend shifts was 4.57±2.75, and the mean number of night shifts was 5.38±4.08. The most common reason for maintaining shift work was “family finances” (76.6%), followed by “self-development” (17.3%) and “social participation” (6.1%) (Table 1).

### Construct validity evaluation

#### Item analysis

The mean scores of the items ranged from 2.07 to 3.63, and no extreme mean values were observed. Skewness and kurtosis met the assumptions of normality. Item-total correlation coefficients ranged from .34 to .66, and no items fell below the threshold of .30. The overall Cronbach’s α for the 42 items was .94, indicating good internal consistency. Therefore, all 42 items were retained for the EFA.

#### Exploratory factor analysis

EFA was conducted three times using data from 210 participants randomly selected from the total sample. Principal component analysis was used for factor extraction, and varimax rotation, an orthogonal rotation method assuming independence among factors, was applied. The KMO measure of sampling adequacy was .90, indicating excellent suitability for factor analysis. Bartlett test of sphericity was statistically significant in all three analyses (first *χ*^2^=4,282.90, second *χ*^2^=2,795.36, third *χ*^2^=2,632.44; all *p*<.001). Item deletion was conducted stepwise when items showed factor loadings below .50 or low communalities that hindered factor interpretation. In the first analysis, nine factors with eigenvalues ≥1.00 were extracted, and 15 items were removed based on the factor loading criteria. In the second analysis, one additional item was deleted. The third analysis identified five factors with eigenvalues ≥1.00 as the final solution. Factor loadings ranged from .50 to .81, communalities ranged from .42 to .76, and the cumulative explained variance was 58.6%. The scree plot also supported a five-factor structure. The factors were labeled as follows: benefits of shift work (eight items, 19.6%), benefits of multiple roles (five items, 12.1%), economic benefits (five items, 10.0%), benefits of family life (five items, 9.5%), and benefits of working (three items, 9.2%) (Table 2).

#### Confirmatory factor analysis

CFA was conducted using data from 200 participants not included in the EFA sample to validate the factor structure of the 26 items identified in the exploratory analysis. In the initial model, several items in the benefits of family life factor showed relatively low factor loadings (.44–.54), and the overall model fit indices (IFI, TLI, and CFI) did not meet recommended thresholds. Therefore, an alternative model excluding the benefits of family life factor was tested. The revised model consisted of four factors and 18 items. Using maximum likelihood estimation, the model showed acceptable fit: *χ*^2^=193.45 (*p*<.001), df=129, *χ*^2^/df=1.50, SRMR=.04, RMSEA=.05 (90% CI, .03–.06), IFI=.96, TLI=.95, and CFI=.96. All indices met recommended criteria for good model fit (Table 3).

#### Convergent and discriminant validity

Convergent validity was supported for all factors. CR values ranged from .81 to .92, exceeding the recommended threshold of .70, and AVE values ranged from .59 to .62, exceeding the recommended threshold of .50. Discriminant validity was confirmed because the confidence intervals of the correlations between factors, calculated as Φ±2×SE, did not include 1.00, indicating adequate distinction among the constructs (Table 4).

### Criterion validity evaluation

Criterion validity was assessed by examining the correlation between the WFES-N developed in this study and the existing WFES. The correlation coefficient between the total scores of the two scales was .76 (*p*<.001). Correlations between subscales ranged from .51 to .94, and all were statistically significant (*p*<.001). These findings indicate that the WFES-N demonstrates substantial criterion validity relative to the established WFES.

### Reliability evaluation

#### Internal consistency reliability

Internal consistency reliability was evaluated for the 21 items retained after CFA by examining item-total correlations and changes in Cronbach’s α when items were deleted. Three items that contributed minimally to reliability improvement were removed, resulting in a final instrument consisting of four factors and 18 items. The overall Cronbach’s α for the scale was .93. The Cronbach’s α values for each factor were .91 for benefits of shift work, .76 for benefits of multiple roles, .73 for economic benefits, and .72 for benefits of working.

#### Test-retest reliability

Test-retest reliability was assessed in 30 married shift-working nurses with a two-week interval between administrations. The ICC was .95 (95% CI, .93–.97), indicating excellent stability of the instrument.

### Finalization of the instrument

**Figure 2.**
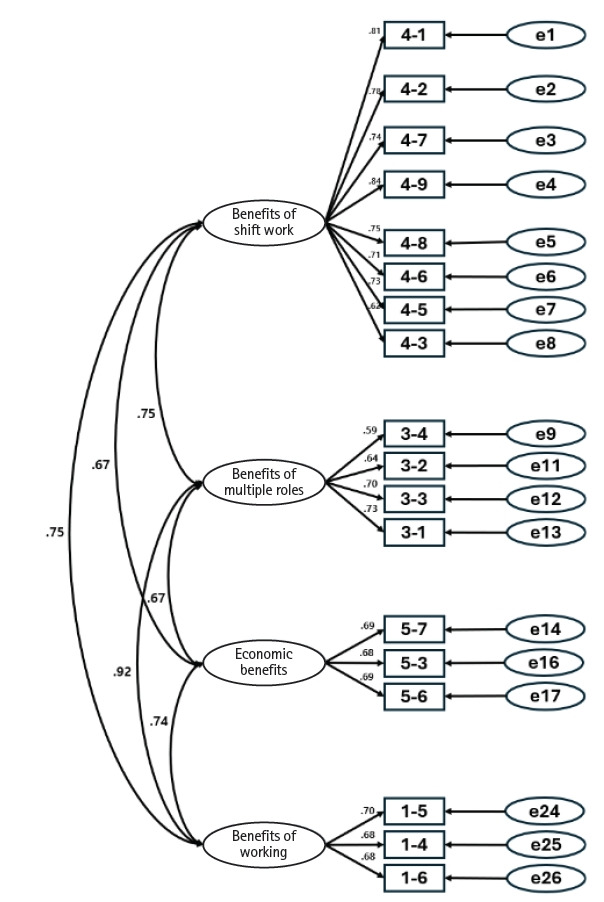
Final model of the work-family enrichment scale.

Following validity and reliability testing, the WFES-N was finalized as a four-factor instrument comprising 18 items measured on a 4-point Likert scale. The subscales consisted of benefits of shift work (eight items; items 1–8), benefits of multiple roles (four items; items 9–12), economic benefits (three items; items 13–15), and benefits of working (three items; items 16–18). Each item is scored from 1 (“strongly disagree”) to 4 (“strongly agree”), yielding a total score ranging from 18 to 72. Higher scores indicate greater work-family enrichment (Figure 2).

## Discussion

This study developed and validated the WFES-N, an instrument designed to systematically assess positive interactions between work and family among married shift-working nurses. Based on the work-family enrichment theory of Greenhaus and Powell [7], the scale operationalizes the concept that resources generated in work and family roles can be transferred across domains to enhance individuals’ quality of life, contextualized for the work and life circumstances of married shift-working nurses. The WFES-N has academic and practical significance in that it simultaneously reflects the unique characteristics of shift work and the life conditions of married nurses.

The results showed that work-family enrichment among married shift-working nurses was structured into four factors: benefits of shift work, benefits of multiple roles, economic benefits, and benefits of working. This finding suggests that work-family enrichment is not a single-dimensional construct but rather a complex experience arising from multiple resource domains, including work schedules, role performance, compensation systems, and workplace experiences [7,18-23]. Whereas previous research has largely interpreted shift work as a burden by focusing on work-family conflict [14,16,32], the present study empirically demonstrates that positive resources can also emerge and be transferred across domains within a shift-work environment.

Examining the factors individually, the benefits of shift work factor reflects the flexibility in time use and opportunities for recovery associated with shift work, which correspond to the concept of temporal resources described by Greenhaus and Powell [7]. Although shift work is often perceived as irregular and demanding, stable shift patterns may provide structural opportunities for schedule adjustment and recovery time [14]. For married nurses, such temporal flexibility can function as a practical resource that supports not only personal physical and psychological recovery but also the flexible management of family responsibilities, such as child-rearing and household duties. These findings are consistent with previous studies reporting that the temporal characteristics of shift work may facilitate work-family enrichment [14,37-39].

The benefits of multiple roles factor reflects the expansion of competencies and adaptive capacity that can arise when individuals perform multiple roles across work and family domains. This dimension corresponds to the concept of skill resources [7]. Skills acquired at work—such as problem-solving ability, communication skills, and time management—may be transferred to family life. Such resource transfer provides an important foundation enabling married nurses to respond effectively to complex role demands across both domains [14]. These findings align with prior research indicating that multiple-role engagement may not only increase the risk of burnout but may also promote personal growth and competence development [25,26].

The economic benefits factor reflects the transfer of financial resources obtained through shift work to improved household financial stability and quality of life. This corresponds to the concept of material resources proposed by Greenhaus and Powell [7]. Compensation elements such as shift allowances and night shift pay appear to function as tangible resources supporting work-family enrichment. In the context of Korean household structures, the professional income of married nurses often plays a significant role in supporting family finances, children’s education, and long-term planning [14]. Thus, financial resources may represent a central pathway through which work-family interactions are strengthened [32,40].

The benefits of working factor reflects the transfer of positive emotional and social resources generated in the workplace to family life. Workplace relationships, collegial cooperation, and organizational support may contribute to emotional stability and positive family relationships [14]. In the context of shift work, such resources may serve as important sources of emotional recovery. These findings are consistent with previous research demonstrating that workplace social resources promote work-family enrichment [21,41,42]. This factor structure highlights both conceptual continuity and meaningful distinctions relative to existing work-family enrichment instruments.

The WFES-N developed in this study showed a strong correlation with the translated WFES (r=.76), and correlations between corresponding subfactors ranged from .51 to .94. These findings suggest that both instruments measure the shared concept of positive resource transfer between work and family, thereby supporting the criterion validity of the WFES-N. However, this correlation does not imply conceptual redundancy. Whereas the WFES measures work-family resource transfer in general working populations at a relatively broad and abstract level, the WFES-N specifies the factor structure by incorporating the work and life contexts of married shift-working nurses. In particular, the benefits of shift work and economic benefits factors explicitly reflect resources derived from concrete working conditions, such as flexible time use and compensation associated with shift and night work. In this regard, the WFES-N maintains conceptual continuity with the WFES while providing a more contextually sensitive assessment of work-family enrichment among married shift-working nurses.

Meanwhile, the benefits of family life factor included in the initial stage was excluded from the final model during CFA. This may reflect structural and cultural contexts in which positive resources generated in family life are less likely to be transferred to the work domain for married shift-working nurses [43]. In Korean society, responsibilities for caregiving and emotional labor within households are still frequently perceived as disproportionately assigned to married women [44]. In such contexts, positive experiences within family life may be internalized as normative role fulfillment rather than recognized as personal resources. These sociocultural norms may therefore hinder the reporting of family-derived resources as independent positive constructs, which may explain why this factor did not converge into a stable structure. Additionally, variation in participants’ child-rearing status and the use of cross-sectional data may have limited the ability to capture family-to-work resource transfer. Future studies should examine this process more closely using longitudinal data and subgroup analyses.

From a theoretical perspective, this study demonstrates that resource transfer pathways in work-family enrichment may be structured differently depending on work arrangements and sociocultural expectations. By applying work-family enrichment theory to the work and life context of married shift-working nurses, this study suggests that work-family enrichment is a context-dependent construct rather than a universal and fixed structure [7,18]. Thus, the study contributes to expanding the applicability of existing theoretical frameworks. From a practical perspective, the WFES-N provides an evaluation tool capable of systematically identifying the positive resources and strengths of married shift-working nurses. The instrument may be useful for improving work systems, managing human resources, and developing nurse support programs. By highlighting not only the negative aspects of shift work but also its positive dimensions, the scale may help inform strength-based interventions aimed at improving quality of life and supporting work-family balance among experienced nurses.

This study has several limitations. First, the gender distribution of participants was heavily skewed toward female married nurses; therefore, caution is required when generalizing the findings to the broader nursing workforce, including male nurses. Second, because the instrument was developed specifically for married shift-working nurses, its applicability to other work arrangements or occupational groups may be limited. Finally, because the benefits of family life factor was excluded from the final model, future research should further examine family-derived resources by developing refined items and conducting longitudinal analyses that consider gender and child-rearing stages.

In conclusion, this study presents the WFES-N as a reliable and valid instrument for systematically evaluating work-family enrichment among married shift-working nurses. The scale provides an important empirical foundation for understanding and supporting the quality of life and work experiences of married nurses. Furthermore, because the instrument reflects characteristics associated with shift work, it may also have potential applicability to married workers in other occupations that involve similar work arrangements.

## Figures and Tables

**Table 1.** General characteristics of the participants (N=410)

Characteristics	Categories	n (%) or mean±SD			t/*χ*^2^	*p*
		Total (n=410)	EFA (n=210)	CFA (n=200)		
Sex	Male	12 (2.9)	6 (2.9)	6 (3.0)	0.01	.932
	Female	398 (97.1)	204 (97.1)	194 (97.0)		
Age (year)		34.65±6.30	34.92±6.87	34.36±0.48	5.08	.166
	<30	88 (21.5)	48 (22.9)	40 (20.0)		
	30–40	240 (58.5)	114 (54.2)	126 (63.0)		
	≥40	82 (20.0)	48 (22.9)	34 (17.0)		
Education level	Bachelor	370 (90.2)	182 (86.7)	188 (94.0)	2.31	.129
	Graduate school	40 (9.8)	28 (13.3)	12 (6.0)		
Current position	Staff nurse	316 (77.1)	156 (74.3)	160 (80.0)	2.63	.269
	Charge nurse	94 (22.9)	54 (25.7)	40 (20.0)		
Nursing experience (year)		8.79±6.18	9.13±6.93	8.45±0.14	4.92	.177
	<10	328 (80.0)	160 (76.2)	168 (84.0)		
	10–15	31 (7.5)	21 (10.0)	10 (5.0)		
	≥15	51 (12.5)	29 (13.8)	22 (11.0)		
Current workplace	General ward	363 (88.5)	187 (89.1)	176 (88.0)	0.14	.987
	Emergency room	22 (5.4)	11 (5.2)	11 (5.5)		
	Intensive care unit	25 (6.1)	12 (5.7)	13 (6.5)		
No. of weekend shifts		4.57±2.75	4.51±2.54	4.64±1.21		
No. of night shifts		5.38±4.08	5.16±4.77	5.61±3.19		
Main reasons for employment	Family finances	314 (76.6)	156 (74.3)	158 (79.0)	3.79	.285
	Self-development	71 (17.3)	28 (13.3)	33 (16.5)		
	Social participation	25 (6.1)	16 (7.6)	9 (4.5)		

CFA: Confirmatory factor analysis; EFA: exploratory factor analysis.

**Table 2.** Results of exploratory factor analysis (N=210)

Items		Communality	Factors				
			1	2	3	4	5
4-1	Shift work provides opportunities to invest time in self-development.	.76	.81	.09	.02	–.06	.29
4-2	Shift work facilitates the division of household tasks.	.68	.78	.15	.08	.01	.22
4-7	Flexible working hours enable the use of personal time before and after commuting.	.73	.77	.06	.13	.27	.19
4-9	Shift work helps manage stress.	.73	.77	.14	.21	.03	.27
4-8	Shift work helps utilize time more freely.	.68	.77	.22	.16	.10	–.02
4-6	Shift work allows time to engage in exercise.	.62	.75	.21	.12	–.09	–.02
4-5	Flexible work adjustments help manage unexpected family situations.	.54	.68	.17	.13	.09	.16
4-3	Shift work allows participation in family events through schedule adjustments.	.48	.56	.22	.29	.13	.09
3-4	Balancing work and family life helps enhance my overall competence.	.56	.18	.69	.12	.13	.16
3-6	Balancing work and family life helps boost my self-esteem.	.57	.14	.68	.29	.07	.02
3-2	Balancing work and family life helps improve problem-solving skills.	.57	.25	.60	.21	.24	.21
3-3	Balancing work and family life helps enhance time management skills.	.59	.34	.59	.22	.11	.26
3-1	Balancing work and family life helps set priorities.	.62	.45	.59	.04	–.09	.25
5-7	Income from work helps with long-term financial planning for the family.	.66	.20	.03	.74	.20	.15
5-5	Income from work fosters a sense of pride.	.62	.08	.22	.72	.01	.21
5-3	Income from work helps support leisure activities.	.51	.14	.25	.62	.19	.03
5-6	Income from work helps with focus on tasks.	.60	.28	.17	.56	.15	.39
5-4	Economic independence reduces dependence on one’s spouse.	.42	.15	.18	.55	.24	–.02
1-7	The educational methods learned at work help with family education (e.g., methods for managing hypertension patients, diabetes patients, breastfeeding methods, etc.)	.63	.09	.10	.03	.77	.14
2-4	Rest at home helps manage stress at work.	.57	–.16	–.09	.25	.68	.04
3-7	Balancing work and family life helps me realize the importance of work and family.	.60	.18	.34	.17	.64	.12
4-4	During shift work, unused daytime hours can be used to handle public office and banking tasks.	.58	.24	.01	.24	.58	–.36
2-6	Emotional support from my family enhances my self-esteem at work.	.46	–.03	.39	.17	.51	.16
1-5	The positive attitude developed at work helps with family life.	.72	.28	.19	.19	.20	.73
1-4	The vitality gained at work helps facilitate family life.	.69	.34	.23	.13	.08	.71
1-6	The leadership experience gained at work helps with decision-making within the family.	.62	.30	.28	.26	.01	.61
Cronbach’s α		.93	.92	.82	.97	.82	.71
Eigenvalue			5.30	3.26	2.84	2.55	2.48
Explained variance (%)			19.6	12.1	10.5	9.5	9.2
Cumulative variance (%)			19.6	31.7	42.2	51.7	60.9
Kaiser-Meyer-Olkin=.90							
Bartlett test of sphericity: *χ*^2^=2,632.44, df=325, *p*<.001							

df: Degree of freedom.

**Table 3.** Result of confirmatory factor analysis (N=200)

Factors	Items	Standardized estimate (β)	SE	Critical ratio	r (*p*)				AVE	CR
					Factor 1	Factor 2	Factor 3	Factor 4		
1. Benefits of shift work	4-1	.81	.07	43.55	1				.59	.92
	4-2	.78	.07	42.69						
	4-7	.74	.06	47.88						
	4-9	.84	.08	35.36						
	4-8	.75	.06	51.30						
	4-6	.71	.07	42.16						
	4-5	.73	.07	39.01						
	4-3	.62	.06	52.89						
2. Benefits of multiple roles	3-4	.59	.05	67.16	.75 (<.001)	1			.60	.86
	3-2	.64	.05	61.96						
	3-3	.70	.05	61.18						
	3-1	.73	.05	62.78						
3. Economic benefits	5-7	.69	.05	69.84	.67 (<.001)	.67 (<.001)	1		.62	.83
	5-3	.68	.05	62.16						
	5-6	.69	.05	63.31						
4. Benefits of working	1-5	.70	.05	59.67	.75 (<.001)	.92 (<.001)	.74 (<.001)	1	.59	.81
	1-4	.68	.06	54.28						
	1-6	.66	.05	64.88						

Ave: Average variance extracted; CR: composite reliability.

**Table 4.** Discriminant validity (N=200)

Factor A	↔	Factor B	Φ	SE	Φ–2×SE	Φ+2×SE
Benefits of shift work	↔	Benefits of multiple roles	.75	.04	.66	.83
Benefits of shift work	↔	Economic benefits	.67	.04	.58	.76
Benefits of working	↔	Benefits of shift work	.75	.05	.65	.85
Benefits of multiple roles	↔	Economic benefits	.67	.03	.61	.72
Benefits of working	↔	Benefits of multiple roles	.92	.03	.84	.98
Benefits of working	↔	Economic benefits	.74	.03	.67	.81
Criteria			Whether [Φ±2×SE] includes 1.00			

Φ: Correlation.

## Supplementary materials

Further details on supplementary materials are presented online (available at https://doi.org/10.4069/whn.2026.02.28).

Supplementary Figure 1. Conceptual framework of the work-family enrichment theory [7]. This figure presents the conceptual framework of work-family enrichment for shift-working married nurses, illustrating the bidirectional transfer of instrumental and affective resources between work and family domains.
