# Supplementary material for: Development and psychometric evaluation of the Work-Family Enrichment Scale for married shift-working nurses in Korea: a methodological study
Source: Womens Health Nurs. 2026 Mar 31;32(1):16–28. doi: 10.4069/whn.2026.02.28 (PMC13071685; doi:10.4069/whn.2026.02.28)
Supplement: Supplementary Figure 1. — Conceptual framework of the work-family enrichment theory [7]. This figure presents the conceptual framework of work-family enrichment for shift-working married nurses, illustrating the bidirectional transfer of instrumental and affective resources between work and family domains. [file whn-2026-02-28-Supplementary-Figure-1.pdf]

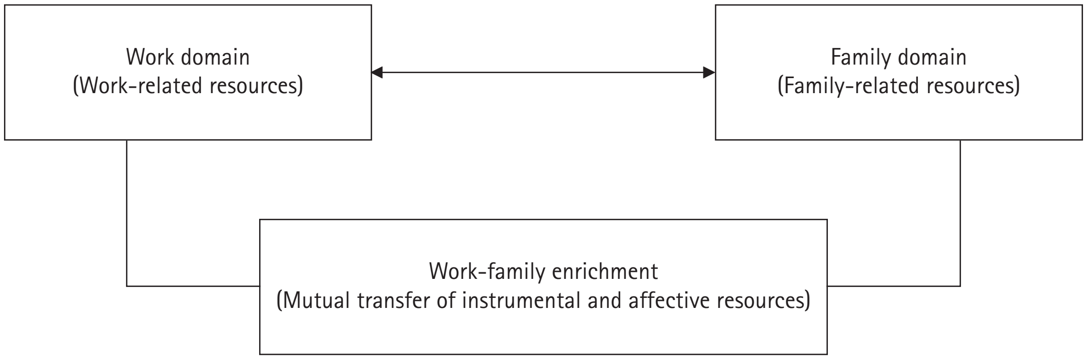

Supplementary Figure 1. Conceptual framework of the work-family enrichment theory [7]. This figure presents the conceptual framework of work-family enrichment for shift-working married nurses, illustrating the bidirectional transfer of instrumental and affective resources between work and family domains.
